# Impact of sustained virologic response on glucose parameters among patients with chronic hepatitis C treated with direct-acting antivirals

**DOI:** 10.20945/2359-4292-2022-0480

**Published:** 2024-05-07

**Authors:** Fábia Benetti, Alexandre de Araújo, Italo de Maman, Cristina Coelho Borges Cheinquer, Fernando Herz Wolff, Hugo Cheinquer

**Affiliations:** 1 Universidade Federal do Rio Grande do Sul Porto Alegre RS Brasil Programa de Pós-graduação em Gastroenterologia e Hepatologia, Universidade Federal do Rio Grande do Sul, Porto Alegre, RS, Brasil; 2 Universidade Federal do Rio Grande do Sul Hospital de Clínicas de Porto Alegre Divisão de Gastroenterologia e Hepatologia Porto Alegre RS Brasil Divisão de Gastroenterologia e Hepatologia do Hospital de Clínicas de Porto Alegre, Universidade Federal do Rio Grande do Sul, Porto Alegre, RS, Brasil; 3 Universidade do Vale do Rio dos Sinos Faculdade de Medicina São Leopoldo RS Brasil Faculdade de Medicina, Universidade do Vale do Rio dos Sinos (Unisinos), São Leopoldo, RS, Brasil

**Keywords:** Glycated hemoglobin, chronic hepatitis C, sustained virologic response, diabetes mellitus, prediabetes

## Abstract

**Objective:**

The aim of this study was to evaluate the glycated hemoglobin (HbA1c) levels before and after sustained virologic response (SVR) and investigate the baseline characteristics associated with improved glycemic control in patients with chronic hepatitis C (CHC) achieving SVR after direct-acting antivirals (DAA) therapy.

**Materials and methods:**

Consecutive adult patients with CHC who achieved SVR after DAA treatment between January 2016 and December 2017 at Hospital de Clínicas de Porto Alegre (RS, Brazil) were prospectively included. Levels of HbA1c were measured up to 24 weeks before DAA therapy and 12 weeks after SVR. Exclusion criteria were decompensated cirrhosis, HIV and/or hepatitis B virus, liver disease of other etiologies, and/or modification of prediabetes/type 2 diabetes mellitus (PDM/T2DM) management. The primary outcome was a comparison of HbA1c levels before and after SVR. Secondary outcomes were the baseline variables associated with improved glycemic control.

**Results:**

The study included 207 patients with a mean age of 60.6±10.7 years, of whom 51.7% were women, 56% had cirrhosis, 37.7% had HCV genotype 3, and 54.5% had baseline T2DM or PDM. The median HbA1c level reduced significantly after SVR (5.5%, interquartile range [IQR] 4.9%-6.3%) compared with baseline (5.7%, IQR 5.3­%-6.7%; p = 0.01). The baseline characteristics associated with improved HbA1c after SVR were cirrhosis, genotype 3, and age ≤ 60 years.

**Conclusion:**

Among patients with CHC, SVR after DAA was associated with HbA1c reduction, particularly in those with cirrhosis, genotype 3, and age ≤ 60 years.

## INTRODUCTION

Hepatitis C virus (HCV) infection is a major public health problem. Indeed, chronic HCV infection is estimated to affect 57 million individuals globally (1). Despite the availability of highly effective direct-acting antivirals (DAAs) since 2014, chronic HCV infection remains one of the main causes of cirrhosis and hepatocellular carcinoma worldwide (1). Several extrahepatic manifestations have been associated with HCV infection, with metabolic disorders of glycemic control being among the most prevalent ones (2). Recent studies have shown that the rates of prediabetes (PDM) and type 2 diabetes mellitus (T2DM) are, respectively, 4 times and 1.5 times higher in persons living with chronic HCV infection compared with noninfected controls (3,4).

Sustained virologic response (SVR), defined as undetectable serum HCV RNA 12 weeks after completion of antiviral therapy, represents cure of HCV infection (5). Large retrospective and prospective cohort studies have shown that SVR significantly improves survival and reduces the risk of disease progression to cirrhosis, hepatic decompensation, and hepatocellular carcinoma (5-9). Also, compelling evidence indicates that SVR obtained with DAAs is associated with improvement in glycemic parameters, both in patients with and without a previous diagnosis of PDM or T2DM. However, glycemic improvement is not observed in all patients, and the baseline characteristics potentially linked to a higher likelihood of glycemic improvement after SVR remain undefined (10-17).

Since 2015, the Brazilian public health system has provided DAA free of charge, with more than 150,000 individuals with HCV treated to date (18). Despite high SVR rates and excellent tolerability with DAAs, few Brazilian studies have assessed the impact of SVR on glucose metabolism (19,20). Thus, the aim of the present study was to compare glycated hemoglobin (HbA1c) levels obtained before and after SVR and investigate potential baseline characteristics associated with this outcome in a cohort of individuals with chronic HCV infection who achieved SVR with DAA therapy at a tertiary referral center in the Southern Region of Brazil.

## MATERIALS AND METHODS

### Study design

This cohort study was carried out at the viral hepatitis outpatient clinic at *Hospital de Clínicas de Porto Alegre* (HCPA), a tertiary referral center of the Brazilian public health system located in Porto Alegre (Rio Grande do Sul, Brazil). The presence of PDM or T2DM was evaluated before treatment and defined according to the American Diabetes Association (ADA) criteria (21). The diagnosis and management of PDM or T2DM were determined by a hepatologist and/or endocrinologist. The serum HbA1c cutoff level for recommendation of treatment with an antidiabetic drug was 7.5%. We used the HbA1c level as a proxy measure of glucose metabolism before and after SVR. No specific recommendation was made regarding diet or physical activity during the study. The presence of cirrhosis was defined using one or more of the following methods: liver biopsy compatible with F4 as defined by the METAVIR system, liver stiffness ≥ 12.5 kPa measured using FibroScan (Echosens, Paris, France), aspartate aminotransferase (AST) to platelet ratio index (APRI score) ≥ 2.0, Fibrosis-4 (FIB-4) index ≥ 3.25, endoscopic finding of gastroesophageal varices, and/or characteristic finding on imaging studies.

### Inclusion and exclusion criteria

The patients were selected according to the following inclusion criteria: age 18 years or above; serum HCV RNA detectable by reverse-transcriptase polymerase chain reaction (RT-PCR) for more than 6 months; documented SVR, defined as an undetectable HCV RNA on RT-PCR at least 12 weeks after the end of a course of all-oral DAA therapy between January 2016 and December 2017; and available HbA1c results at least 24 weeks before the start of DAA therapy and 12 weeks after SVR. The DAA regimens were prescribed according to the Clinical Protocol and Therapeutic Guidelines for Hepatitis C and Coinfections issued by the Brazilian Ministry of Health at the time of the study (22); the recommendations included the following interferon-free therapeutic options: sofosbuvir plus simeprevir (for HCV genotype 1), sofosbuvir plus ribavirin (HCV genotype 2), and sofosbuvir plus daclatasvir (HCV genotype 3).

The exclusion criteria included a history of liver decompensation, coinfection with the human immunodeficiency virus (HIV) and/or hepatitis B virus (HBV), liver disease of other etiologies, modification of clinical or pharmacological management of PDM or T2DM or introduction of agents that could impact glycemic homeostasis during the study period according to a review of medical record or patient interview. Also, patients with worsening glycemic control who required adjustment of antidiabetic drugs were excluded. Patients were also excluded when having any condition that could impact HbA1c measurement, including pregnancy, hemodialysis, recent blood loss, transfusion, erythropoietin therapy, hemoglobinopathies, or glucose-6-phosphate dehydrogenase deficiency.

### Statistical analysis

Numerical data are presented as mean ± standard deviation or median (interquartile range [IQR]), while categorical variables are presented as frequencies (percentages). Numerical variables were not normally distributed, as verified by the Kolmogorov-Smirnov test; therefore, quantitative variables were compared using Mann-Whitney or Wilcoxon signed-rank nonparametric tests, as appropriate. Categorical variables were compared using the chi-square test or Fisher's exact test, as fit, with adjusted residual analysis applied to detect categories appearing more frequently than expected. The statistical analyses were performed using SPSS for Windows, version 22.0 (IBM Corp., Armonk, NY, USA). A p value lower than 0.05 was deemed statistically significant. Considering a mean reduction of 0.5 mg/dL in HbA1c level after SVR (23), an alpha error of 5%, and a statistical power of 80%, the sample size (calculated using WINPEPI, version 11.65) was estimated to be at least 64 individuals.

### Ethical aspects

The protocol of the study was approved by the ethics and research committee of the local institution. The study was conducted in accordance with the ethical principles of the Helsinki Declaration and was registered in Plataforma Brazil under the Certificate of Presentation for Ethical Appreciation number 85706418.6.0000.5327.

## RESULTS

**Figure 1:**
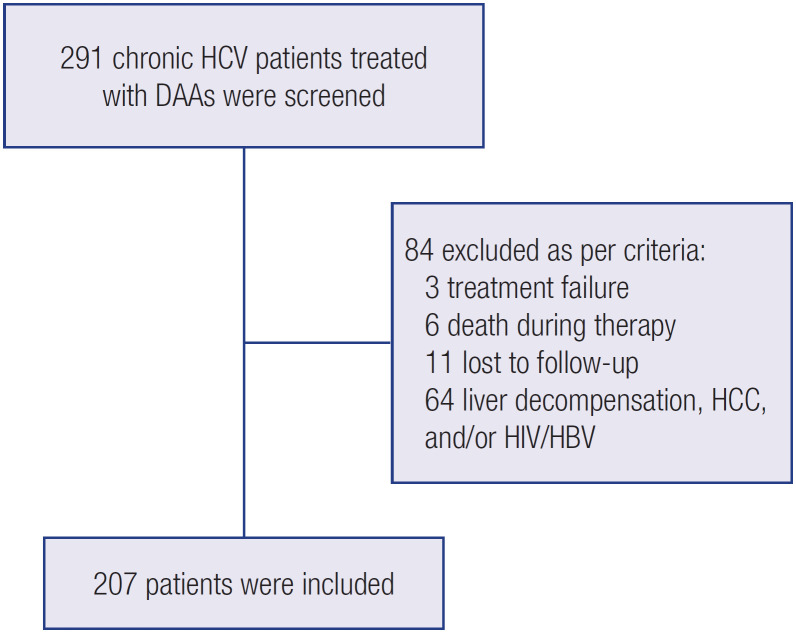
Flow chart of patients selected to study.

Of 291 patients screened, 207 were included in the study (Figure 1). The mean age of the participants was 60.7 ± 10.7 years (range 19-91 years), and 119 (57.5%) were older than 60 years. Cirrhosis was diagnosed in 116 (56%) individuals. Table 1 summarizes the main baseline characteristics of the study cohort.

**Table 1:** Baseline characteristics of the 207 study patients

		Overall n = 207	T2DM or PDM n = 91	Normoglycemic n = 116
Age (years) mean SD		60.7 ± 10.7	61.6 ± 9.5	59.9 ± 11.5
Male gender – n (%)		100 (48.3%)	45 (49.4%)	55 (47.4%)
Body mass index* – n (%)				
	<25	44/142 (31%)	19/63 (30.2%)	25/79 (31.7%)
	≥25	98/142 (69%)	44/63 (69.8%)	54/79 (68.3%)
HCV genotype – n (%)				
	1	124 (59.9%)	51 (56%)	73 (62.9%)
	2	5 (2.4%)	4 (4%)	1 (0.8%)
	3	78 (37.7%)	36 (40%)	42 (36.3%)
Cirrhosis – n (%)				
	No	91 (44%)	34 (37.3%)	57 (49.1%)
	Yes	116 (56%)	57 (62.7%)	59 (50.9%)
Fasting plasma glucose (mg/dL) – median (IQR)		98 (90-115)	118 (103-142)	93 (87-98)
HbA1c (%) mean ± SD		5.7 (5.3-6.7)	6.4 (5.7-7.2)+	5.2 (4.5-5.5)+
Esophageal varices – n (%)		92 (44.4%)	44 (48.3%)	48 (41.4%)

Note: Data are presented as n (%), mean ± SD or median (IQR); T2DM: type 2 diabetes mellitus; PDM: pre-diabetes.

* Data available in 142 of the 207 patients.

+ p < 0.05.

**Figure 2:**
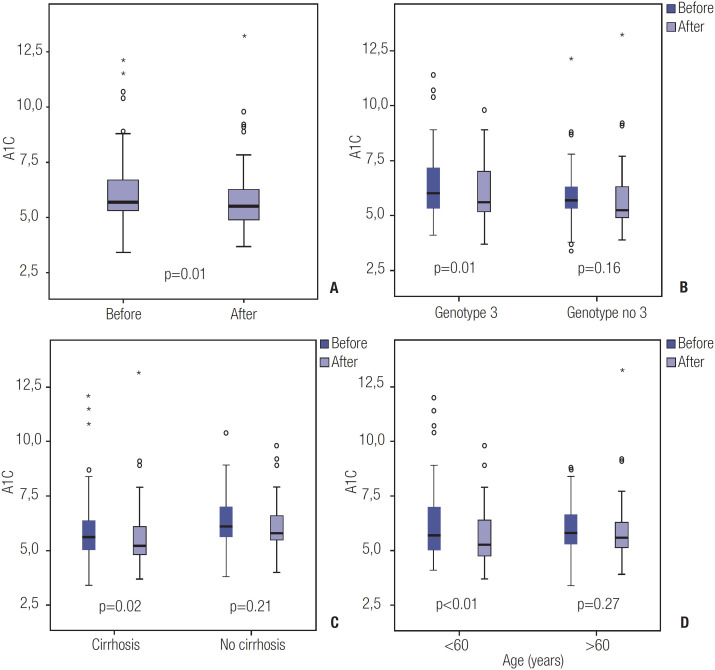
Overall HBA1C results at baseline and after SVR (**A**); HBA1C results in patients with and without genotype 3 (**B**), with and without cirrhosis (**C**) and according to age (**D**).

A comparative analysis of HbA1c levels before and after SVR is summarized in Table 2. The median HbA1c level improved significantly after SVR (5.7%, IQR 5.3%-6.7%) compared with baseline (5.5%, IQR 4.9%-6.3%; p = 0.01) (Figure 1). The following baseline variables were associated with significant improvement in glycemic control after SVR: presence of cirrhosis (p = 0.02), genotype 3 (p = 0.01), and age ≤ 60 years (p = 0.004) (Table 2 and Figure 2). The improvement in HbA1c level after SVR among normoglycemic individuals and patients with impaired glucose metabolism is depicted in Table 3.

**Table 2:** Glycated hemoglobin (HbA1c) before and after HCV therapy according to selected baseline characteristics

		Baseline HbA1c Median % (IQR)	HbA1c after SVR Median % (IQR)	p value
Overall (n = 207)		5.7 (5.3-6.7)	5.5 (4.9-6.3)	0.01
Cirrhosis				
	Yes (n = 116)	5.6 (5-6.5)	5.3 (4.8-6.2)	0.02
	No (n = 91)	6.1 (5.5-7.1)	5.8 (5.5-6.7)	0.21
Genotype				
	3 (n = 78)	6.1 (5.3-7.2)	5.6 (5.1-7.1)	0.01
	Non-3 (n = 129)	5.7 (5.3-6.5)	5.3 (4.9-6.3)	0.16
Age ≤ 60 years				
	Yes (n = 119)	5.8 (5.3-6.7)	5.6 (5.1-6.3)	0.27
	No (n = 88)	5.7 (5-7.2)	5.3 (4.7-6.7)	<0.01
BMI ≥ 25 kg/m^2^*				
	Yes (n = 98)	5.9 (5.35-7.12)	5.6 (4.9-7.22)	0.06
	No (n = 44)	5.65 (5.27-6.77)	5.75 (5.17-6.82)	0.71

* Data available in 142 of the 207 patients.

**Table 3:** Improvement in HBA1C before and after SVR among HCV patients with or without impaired glucose metabolism

Pre-diabetes or type-2 diabetes mellitus	Baseline HBA1C Median % (IQR)	HBA1C after SVR Median % (IQR)	p value
No (n = 116)	5.2 (4.5-5.5)	4.9 (4.5-5.3)	0.05
Yes (n = 91)	6.4 (5.7-7.2)	5.9 (5.3-7.2)	0.03

A total of 91 patients had T2DM or PDM, of whom 46 were treated at baseline with oral hypoglycemic drugs or insulin. Recurrent hypoglycemia occurred in 3 of 13 patients treated with insulin after SVR (23%), and the insulin dose was reduced in these patients. The mean HbA1c level among 45 patients who did not receive antidiabetic drugs was 6.01%.

**Figure 3:**
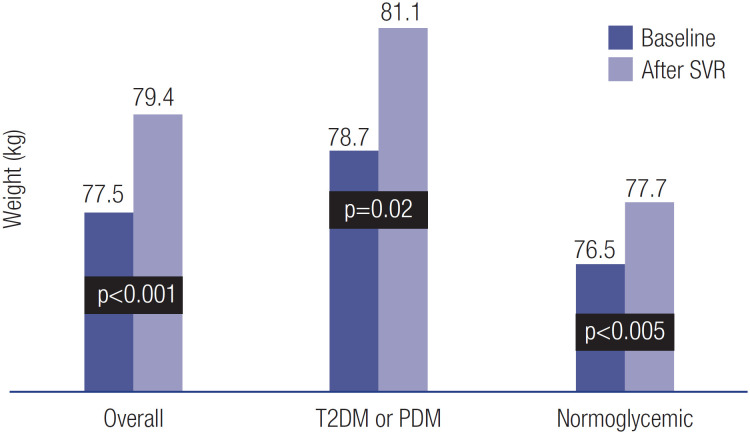
Patients weight at baseline and after SVR.

The improvement in HbA1c level occurred despite weight gain observed after SVR (Figure 3).

## DISCUSSION

The present study evaluated patients with compensated chronic hepatitis C and SVR after DAA therapy. Most patients had HCV genotype 1 or 3, and almost half of them had cirrhosis, clinically significant portal hypertension, and normoglycemia or PDM/T2DM. The HbA1c level improved significantly after HCV clearance, particularly among patients with cirrhosis, HCV genotype 3, or age ≤ 60 years.

Infection by HCV is a systemic disease with a wide spectrum of clinical presentation, including hepatic and extrahepatic manifestations (2). Disturbance of glucose metabolism is one of the most frequent extrahepatic manifestations of chronic HCV infection, with PDM occurring four times more frequently in patients with HCV (4). Regarding T2DM, a recent meta-analysis found a 15% prevalence of T2DM among 61,843 individuals infected with HCV, compared with 10% among 202,130 controls not infected with HCV, indicating a risk of T2DM 1.5 times higher in patients with HCV (odds ratio 1.58, 95% confidence interval 1.30-1.86) (3).

The emergence of DAA therapy has revolutionized the management of HCV infection, with remarkable safety and efficacy reports in clinical studies and real-life cohorts (22,24-32). Achievement of SVR has a proven beneficial impact on liver disease outcomes, reducing the risk of progression to cirrhosis, hepatic decompensation, hepatocellular carcinoma, liver transplantation, and death (5-9). However, the impact of HCV eradication on glucose metabolism disturbance is not so well defined (10-17).

Recent prospective studies suggest that HCV clearance by DAAs improves glucose control in patients with and without T2DM and prevents the occurrence of T2DM after SVR when these patients are compared with untreated controls or with individuals who fail to achieve SVR (11,25,28-30). We found similar results in the present study, with a significant overall decrease in median HbA1c level from 5.7% at baseline to 5.5% at 12 weeks after SVR.

In our cohort, the beneficial impact of SVR on HbA1c level was more relevant among patients with a previous diagnosis of PDM or T2DM. Indeed, our data showed a higher improvement in glucose control among patients with T2DM or PDM (HbA1c levels of 6.4% *versus* 5.9% before and after DAA therapy, respectively; p = 0.03) compared with those without glucose disturbance (HbA1c levels of 5.2% *versus* 4.9% before and after DAA therapy, respectively, p = 0.05). The improvement in glycemic control was significant in both groups (with and without abnormal glucose control). However, the difference observed regarding HbA1c levels before and after SVR in individuals without PDM or T2DM was only 0.3%, which may not be clinically significant. On the other hand, the improvement in HbA1c levels before and after SVR noted among patients with PDM or T2DM was 0.5%, which by itself is considered potentially meaningful at a clinical level. A similar study conducted by Takahashi and cols. followed 272 patients with HCV infection who achieved SVR with DAA therapy; the authors found greater improvement in glucose homeostasis among 55 patients with T2DM at baseline compared with patients without T2DM, with a significant reduction in median HbA1c level from 7.2% at baseline to 6.8% at SVR (16).

In the present study, the improvement in glycemic parameters was only significant in patients with cirrhosis, HCV genotype 3, and/or age below 60 years. The greater benefit of SVR on glucose parameters observed among patients with cirrhosis could be related to the fact that these patients are more likely to have a higher degree of insulin resistance. Thus, HCV eradication could potentially have a greater impact on glucose parameters among patients with more advanced disease progression. Similar results have been reported by Cacciola and cols., who followed patients with chronic HCV infection and T2DM treated with DAA therapy and found significant improvement in long-term glucose control among those with advanced fibrosis or cirrhosis (10). Salomone and cols. evaluated prospectively 32 patients with chronic HCV and compensated cirrhosis who had SVR after DAA therapy and no T2DM and found significant improvement in HbA1c level (from 6.1 ± 0.2% before treatment to 5.7 ± 0.3% after SVR; p < 0.001) (14).

Interestingly, our study found a significant improvement in HbA1c level in a subgroup of patients with HCV genotype 3 compared with those with genotypes 1 and 2. Kanwal and cols. studied 110,484 patients with chronic HCV infection and found a 31% higher risk of cirrhosis among those infected with genotype 3 compared with genotype 1 (33). This finding could be related to the fact that genotype 3 leads to viral-related hepatic steatosis independent of metabolic factors and has a higher chance of being resolved after HCV eradication. However, it seems that the proportion of patients with HCV infection and glycemic disorders is similar among all genotypes. Indeed, Rajewski and cols. evaluated 2,898 patients with HCV infection and found that T2DM was significantly more common among those with more advanced liver fibrosis, increased age, and/or male sex, but not in those with HCV genotype 3 (34). Insulin resistance before and after SVR was not assessed in the present study. Nevertheless, a previous study conducted at our center evaluating 44 treatment-naïve patients with chronic hepatitis C found no significant difference in proportions of insulin resistance above 2.0 as measured by the homeostatic model assessment of insulin resistance (HOMA-IR) between patients with HCV genotypes 1 or 3 (65% *versus* 57%, respectively p = 0.81) (25).

Moreover, we found improved glycemic control in patients aged ≤60 years. Metabolic disturbances are more prevalent in older individuals, and the HCV clearance could have had a higher impact on improving viral-induced glycemic disorders in younger patients.

Several potential limitations should be taken into account when the results of the present study are interpreted. These include the single measurement of HbA1c 24 weeks after HCV therapy with DAA and the lack of longer follow-up, measurement of insulin resistance, or multiple evaluations of glucose control over time to assess the sustainability of the response to HCV clearance with DAAs. Strengths of the present study include the prospective inclusion of all consecutive HCV individuals treated with DAA who achieved SVR, with or without PDM/T2DM, followed for at least 24 weeks after therapy with measurement of HbA1c before and after treatment, and the exclusion of conditions that could interfere with glucose homeostasis. Furthermore, the improvement in HbA1c level occurred despite weight gain after SVR.

The issue of increased body weight after SVR has been noted elsewhere. There is a lack of consensus regarding the exact reason for this occurrence, which has been usually related to the general clinical improvement commonly seen after HCV elimination (35,36). Also, the insulin dose requirement was reduced in 23% of the patients with T2DM on insulin treatment after SVR. The results of the present study indicate that HCV clearance improves glycemic control particularly among individuals with cirrhosis, HCV genotype 3, and/or age ≤ 60 years. Future prospective studies may help determine if these variables are indeed predictive of a higher chance of metabolic improvement in glucose parameters among patients with chronic HCV who achieve SVR after DAA therapy.
